# Celluloid Base

**Published:** 1872-04

**Authors:** 


### Celluloid Base.

We are very often asked, "What about celluloid base?" "Is it as good as rubber?" Yes; we think better. "Can the springing be prevented?" Yes; many who are making it do prevent it, and have no trouble with it in this direction, and in this matter what some can do others may and will if they employ the same means. All persons can not with the same ease accomplish desirable results. Some are unskillful, others are awkward, others are careless, others are impatient—lack careful study, industrious perseverance. All these classes, and they are each quite large, will make failures with anything for a long time. One says, "Does it work as easily as rubber?" We are sorry to say it does; it even requires less skill.

"Is the camphor easily removed from it?" Yes, very, by using the right method.

"Do we recommend calluloid?" Yes, if you must use some contemptible stuff for plates use this rather than rubber, which is an abomination and a curse to humanity.

